# A single proteolytic cleavage within the lower hinge of trastuzumab reduces immune effector function and *in vivo *efficacy

**DOI:** 10.1186/bcr3240

**Published:** 2012-08-08

**Authors:** Xuejun Fan, Randall J Brezski, Ming Fa, Hui Deng, Allison Oberholtzer, Anneliese Gonzalez, William P Dubinsky, William R Strohl, Robert E Jordan, Ningyan Zhang, Zhiqiang An

**Affiliations:** 1 Texas Therapeutics Institute, Brown Foundation Institute of Molecular Medicine, University of Texas Health Science Center at Houston, 1825 Pressler Street, Houston, TX 77030, USA; 2 Biologics Research, Janssen R&D, LLC, 145 King of Prussia Road, Radnor, PA 19087, USA; 3 Division of Oncology, Department of Internal Medicine, University of Texas Health Science Center at Houston, 6431 Fannin Street, Houston, TX 77030, USA; 4 School of Dentistry, University of Texas Health Science Center at Houston, 7500 Cambridge Street, Houston, TX 77054, USA

## Abstract

**Introduction:**

Recent studies reported that human IgG antibodies are susceptible to specific proteolytic cleavage in their lower hinge region, and the hinge cleavage results in a loss of Fc-mediated effector functions. Trastuzumab is a humanized IgG_1 _therapeutic monoclonal antibody for the treatment of HER2-overexpressing breast cancers, and its mechanisms of action consist of inhibition of HER2 signaling and Fc-mediated antibody-dependent cellular cytotoxicity (ADCC). The objective of this study is to investigate the potential effect of proteinase hinge cleavage on the efficacy of trastuzumab using both a breast cancer cell culture method and an *in vivo *mouse xenograft tumor model.

**Methods:**

Trastuzumab antibody was incubated with a panel of human matrix metalloproteinases, and proteolytic cleavage in the lower hinge region was detected using both western blotting and mass spectrometry. Single hinge cleaved trastuzumab (scIgG-T) was purified and evaluated for its ability to mediate ADCC and inhibition of breast cancer cell proliferation *in vitro *as well as anti-tumor efficacy in the mouse xenograft tumor model. Infiltrated immune cells were detected in tumor tissues by immunohistochemistry.

**Results:**

scIgG-T retains HER2 antigen binding activity and inhibits HER2-mediated downstream signaling and cell proliferation *in vitro *when compared with the intact trastuzumab. However, scIgG-T lost Fc-mediated ADCC activity *in vitro*, and had significantly reduced anti-tumor efficacy in a mouse xenograft tumor model. Immunohistochemistry showed reduced immune cell infiltration in tumor tissues treated with scIgG-T when compared with those treated with the intact trastuzumab, which is consistent with the decreased ADCC mediated by scIgG-T *in vitro*.

**Conclusion:**

Trastuzumab can be cleaved by matrix metalloproteinases within the lower hinge. scIgG-T exhibited a significantly reduced anti-tumor efficacy *in vivo *due to the weakened immune effector function such as ADCC. The results suggest that the lower hinge cleavage of trastuzumab can occur in the tumor microenvironment where matrix metalloproteinases often have high levels of expression and scIgG-T might compromise its anti-tumor efficacy in the clinic. However, further studies are needed to validate these hypotheses in the clinical setting.

## Introduction

Trastuzumab is a humanized IgG_1 _monoclonal antibody for the treatment of primary and metastatic breast cancers that overexpress HER2 [1]. Both antigen engagement by the Fab region, which results in HER2 signaling inhibition, as well as induction of immune effector functions such as antibody-dependent cellular cytotoxicity (ADCC) mediated by the Fc region play important roles in the mechanisms of action of trastuzumab [2-4]. Despite the clinical success of trastuzumab in treating high HER2 breast cancers, primary and acquired resistance to the therapy is widespread in the clinic [5]. Previous studies on resistance to trastuzumab have focused in large part on cell signaling escape mechanisms. These studies have included loss of phosphatase and tensin homolog function, gain of function mutations in signaling molecules such as phosphatidylinositol 3-kinase and protein kinase B (AKT) [6,7], activation of HER family member receptors epidermal growth factor receptor and HER3 [8], and upregulation of other receptor tyrosine kinases such as insulin-like growth factor 1 receptor [9], hepatocyte growth factor receptor (cMET) [10], and ephrin-A family tyrosine kinase receptor 2 [11].

IgG antibody is known to be susceptible to specific cleavage within the hinge region by proteinases *in vitro *[12,13]. Extracellular proteinases secreted by certain human bacterial pathogens can cleave human IgGs within the lower hinge region, and these proteinases are suggested to function as virulence factors by evading the host immune response to bacterial infections [14-17]. Recent reports have also demonstrated that certain human matrix metalloproteinases (MMP-3, MMP-7, MMP-9, MMP-12 and MMP-13) can catalyze a single-strand cleavage of human IgG_1 _antibodies in the lower hinge region *in vitro *[15,18], although the rate of cleavage varies among the different MMPs. Purified single-cleaved IgG_1 _antibodies were shown to have substantially depressed immune effector functions such as ADCC and complement-dependent cytotoxicity [18-20]. The loss of antibody Fc effector function was correlated with a decreased binding to Fcγ receptors that are expressed on immune effector cells such as natural killer (NK) cells and monocytes [18,21]. Since ADCC is considered one of the key mechanisms of action for trastuzumab [3,22-26], factors that compromise Fc-mediated immune functions of trastuzumab are expected to decrease its efficacy.

The study described in this report investigated the impact of trastuzumab hinge cleavage on its anti-HER2 signaling function and anti-tumor efficacy *in vitro *and *in vivo*. The results demonstrated that single cleavage of trastuzumab within the lower hinge severely impaired Fc-mediated immune effector cell function *in vitro *and resulted in significantly reduced anti-cancer efficacy *in vivo*. These findings underscore the potential effects of proteolytic hinge cleavage of trastuzumab and other therapeutic antibodies in the tumor microenvironment by compromising their clinical efficacy.

## Materials and methods

### Enzymes, antibodies and cell lines

Trastuzumab was purchased from a specialty pharmacy. Single hinge cleaved trastuzumab (scIgG-T) was prepared by enzymatic digestion with a bacterial proteinase, IgG-degrading enzyme S (IdeS), as previously described [15,18]. Recombinant IdeS was expressed in *Escherichia coli *and purchased from Genovis AB (Lund, Sweden). The isotype control monoclonal antibody (human IgG_1_) was expressed at Janssen R&D, LLC (Radnor, PA, USA). The cancer cell lines SKOV-3 and BT474 were obtained from American Type Culture Collection (Manassas, VA, USA), and were grown in RPMI 1640 media supplemented with 10% fetal bovine serum, 2 mM glutamine, 50 units/ml penicillin, and 50 µg/ml streptomycin in an incubator with 5% CO_2 _at 37°C. Antibodies for total HER2, pHER2 (Y1248), pHER3 (Y1289), pEGFR (Y1068), pAKT (S473), and pErk1/2 (T202/Y204) were purchased from Epitomics (Burlingame, CA, USA).

### Capillary gel electrophoresis

The method used for antibody hinge cleavage and gel analysis were reported previously [15]. Briefly, trastuzumab was subjected to proteolysis by recombinant human MMPs or the bacterial protease IdeS in Tris-buffered saline plus calcium chloride. Electrophoresis separation was performed using an Agilent 2100 microfluidics-based Bioanalyzer (Agilent Technologies, Santa Clara, CA, USA). Proteins were applied at 1 mg/ml in Tris-buffered saline and run on Agilent 230 chips following the manufacturer's protocol [27]. Trastuzumab digests were run under nonreducing conditions to best visualize the single-cleaved products.

### Flow cytometry

Binding of trastuzumab and scIgG-T on HER2-expressing breast cancer cells was measured using a Guava easyCyte HT instrument based on the manufacturer's instructions (Millipore, Danvers, MA, USA). Briefly, 5×10^5 ^cells were dispensed in 100 µl aliquots and trastuzumab as primary antibody was added for 1 hour at 25°C, followed by the addition of phycoerythrin-conjugated anti-human-Fc (Jackson ImmuneResearch Laboratories, Inc., West Grove, PA, USA). After washing with PBS buffer, the cells were analyzed for fluorescence intensity and human isotype IgG_1 _antibody was used as the reference sample control.

### Fcγ receptor binding assays

Binding of Fcγ receptors with antibodies was determined by ELISA as reported previously [28]. Fcγ receptors were from R&D Systems (Minneapolis, MN, USA). The fluorescence signal was detected at excitation 340 nm and emission 460 nm using a plate reader (Molecular Devices, Sunnyvale, CA, USA).

### Cell lysate preparation from tumor tissues

Tumor tissues from mouse xenograft studies or breast cancer patients were homogenized using gentle MACS (Miltenyi Biotec, Bergisch Gladbach, Germany) according to the manufacturer's procedure in a cell lysis buffer in the presence of proteinase inhibitor cocktails (Calbiochem, San Diego, CA, USA). Trastuzumab was affinity enriched from the tissue lysate using HER2 extracellular domain protein (Sino Biologicals, Beijing, China) and used for SDS-PAGE and western blotting analysis.

### Western blotting

Cell lysates prepared from cell culture or tumor tissues were subjected to SDS-PAGE separation. Gels were either stained with Coomassie blue (Bio-Rad, CA, USA) or transferred to a polyvinylidene fluoride membrane by standard procedures. Membranes were blotted using a goat-anti-human Fc-horseradish peroxidase (Sigma-Aldrich, St. Louis, MO, USA) and images were detected with FluorChem M imager (Cell BioSciences, Santa Clara, CA, USA) using enhanced chemiluminescence substrate (GE Healthcare, Piscataway, NJ, USA).

### Antibody-dependent cellular cytotoxicity assay

ADCC activities were assayed as described previously [29] using the xCELLigence instrument (Roche, Mannheim, Germany). Briefly, the high HER2 expression cancer cell line SKOV-3 was seeded in E-plate 96 (ACEA Biosciences, Inc., San Diego, CA, USA), followed by the addition of human peripheral blood mononuclear cells (PBMC) cells as immune effector cells. Human PBMC were isolated from fresh blood samples collected from consented healthy volunteers using procedures reported previously [18]. Antibodies were added in three-fold titration starting from 5 µg/ml, and the cell index (cell growth) was monitored continuously for 2 days. Cancer cells with PBMC only were used as the control group and the ratio of effector cells to target cells was 50:1. The cell index recorded after 24 hours of treatment with trastuzumab and scIgG-T was used to calculate the percentage of cell lysis using the formula:

$$\left(\text{Cell}\;\text{index}\;\text{of}\;\text{control}\;\text{group}-\text{cell}\;\text{index}\;\text{of}\;\text{treatment}\;\text{group}\right)/\text{cell}\;\text{index}\;\text{of}\;\text{control}\;\text{group}\times \text{1}00$$

Each treatment contains three replicates and the standard deviation is shown in the plots.

### Cell proliferation assay

Inhibition of breast cancer cell proliferation by trastuzumab and scIgG-T was measured based on a reported procedure [29]. Briefly, BT474 cells (5,000 cells/well) were seeded in a 96-well plate and cultured overnight in RPMI medium with 10% fetal bovine serum at 37°C and 5% CO_2_. Treatment antibodies were added into the cell culture in a series of concentrations (0 to 10 µg/ml) and continued incubation for 72 hours. Alamar Blue (Invitrogen, Carlsbad, CA, USA) was added according to the manufacturer's instructions and fluorescence signal was read using a SpectraMax M4 plate reader (Molecular Devices) using excitation at 535 nm and emission at 590 nm.

### Mouse xenograft tumor model

Mouse xenograft studies were carried out in accordance with the animal care and use guidelines and the protocol was approved by the Animal Welfare Committee of the University of Texas Medical School at Houston. HER2-overexpressing BT474 breast cancer cells were used in the xenograft model as previously described [29]. Immunodeficient nu/nu mice from a homogeneous BALB/c background were from Charles River Laboratories (Wilmington, MA, USA). When tumors reached 50 to 100 mm^3^, mice were randomized into treatment groups with five mice per group. Antibodies were administered weekly intraperitoneally at 5 mg/kg dosing level for 6 weeks. Tumor size was measured and recorded every 3 or 4 days using a Vernier scale caliper. Tumor tissue and sera were collected and stored at -80°C until analysis.

### Immunohistochemistry

Xenograft tumor tissues were excised freshly and fixed in 4% paraformaldehyde and embedded in paraffin. Serial 4-μm thick sections were made for staining. Anti-mouse integrin αM/CD11b and F4/80 antibodies (R&D Systems) were applied as primary antibody, and positive staining was visualized using a three-step staining procedure with an Elite ABC kit (Vector Laboratories, Peterborough, UK) and counterstained with hematoxylin (Vector Laboratories). Six tumor tissue sections (*n *= 6) were made from each treatment group and the tissue slides were viewed using Nikon Eclipse E200 microscopy and the entire tumor sections were scanned at 10× (magnification = 10×10) to identify all positive stained cells. Representative images from each treatment group were taken under ×40 magnification. The average number of immune cell infiltration per tumor slide under ×40 magnification was plotted for each treatment groups and standard error was calculated among the six tissue slides.

### Mass spectrometry analysis

Mass spectral analyses were performed in the Translational Proteomics Core at University of Texas Health Science Center at Houston. Protein bands were excised and *in-gel *digestion was performed as previously described [30]. Tryptic digested proteins were taken to dryness in a Thermo SpeedVac and dissolved in 20 µl of 2% acetonitrile, 0.1% formic acid (solvent A). Aliquots of the digest were analyzed by liquid chromatography/mass spectrometry (MS)/MS on an Agilent 6538 UHD Accurate-Mass Quadrupole Time-of-Flight mass spectrometer equipped with an Agilent 1260 nanoLC system. The reverse-phase chromatography was performed on an Agilent High Capacity Chip (143 mm) using solvent A as the initial mobile phase and varying percentages of solvent B (90% acetonitrile, 0.1% formic acid) to constitute a gradient elution. Electrospray ionization was operated at the spray voltage of 1.75 kV. Mass spectral data were extracted with the MassHunter Quantitative Analysis package (Agilent Technologies, Inc., Santa Clara, CA USA) and peptides were identified from MS/MS spectra with MASCOT (Matrix Science Inc., Boston, MA, USA). The MASCOT search was performed with a peptide tolerance of 5 ppm and an MS/MS tolerance of 0.05 Da, fixed modification with carbamidomethyl and variable methionine oxidation. Identification of nontryptic fragments was performed manually with an initial search on the basis of predicted peptide masses of all possible fragments resulting from novel cleavage sites in the hinge region. The MS/MS spectra of suspect peaks were verified by manual *de novo *peptide sequencing to confirm their identities.

### Patient samples

Individuals enrolled in the study protocol gave informed consent and all procedures and the protocol were reviewed and approved by the institutional review board of the University of Texas Medical School at Houston. Breast tumor tissue and adjacent normal tissues 2 cm from the tumor sites were obtained from breast cancer patients immediately following surgery by a certified pathologist and were snap-frozen in liquid nitrogen until analysis.

### Detection of MMP expression in human breast cancer tissues

MMP expression was measured using the Quantibody reverse-phase human MMP array kit according to the manufacturer's instructions (RayBiotech, Norcross, GA, USA). Fluorescence images were detected using a GenePix 4100A Scanner, and data were analyzed using the QAH-MMP-1 GAL software based on the instruction provided by the array manufacturer.

### Statistical analysis

Where appropriate, statistical analysis was performed using paired Student's *t *test. *P *< 0.05 between treatment groups is considered significantly different.

## Results

### Trastuzumab can be cleaved within the lower hinge *in vitro *by human MMPs

**Figure 1:**
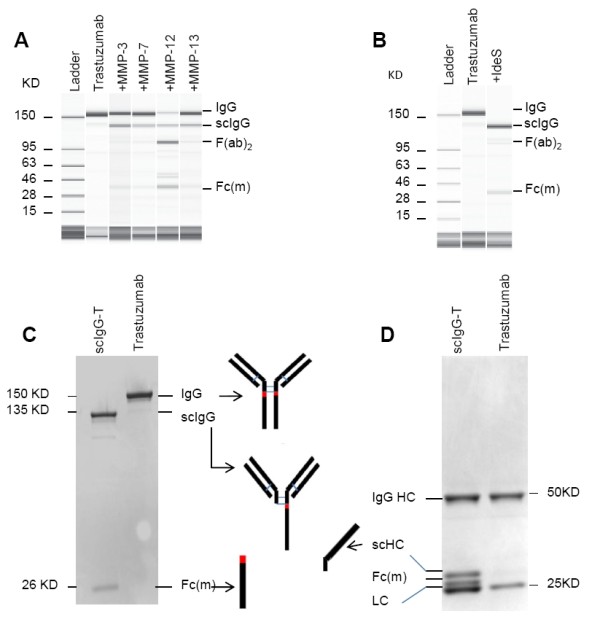
**Detection of trastuzumab proteolytic cleavage *in vitro***. **(A) **Capillary electropherograms (CEs) of trastuzumab and matrix metalloproteinase (MMP)-digested trastuzumab under nonreducing and denatured running conditions. **(B) **CE of trastuzumab and IgG-degrading enzyme S (IdeS)-digested single hinge cleaved trastuzumab (scIgG-T). **(C) **Purified scIgG-T under nonreducing and denaturing running conditions and stained with Coomassie blue. **(D) **Trastuzumab and scIgG-T under reducing and denaturing running conditions and stained with Coomassie blue. Fab, fragment antigen binding; Fc_(m)_, Fc monomer; HC, heavy chain; LC, light chain.

An increased expression of human MMPs has been reported in the tumor microenvironment of various cancer types, including breast cancer [31]. To investigate whether MMPs catalyze specific single hinge cleavage of trastuzumab, a panel of four human MMPs were individually incubated with trastuzumab *in vitro*, and hinge cleavage was detected using a capillary electrophoresis separation system. As shown in Figure 1A, trastuzumab was readily cleaved at the hinge by all four MMPs tested, as indicated by the production of scIgG-T. Based on previous studies of other IgG_1 _antibodies [18,32], IdeS produced by *Streptococcus pyogenes *can also cleave human IgGs at the hinge region. We treated trastuzumab with IdeS and, as expected, IdeS efficiently cleaved trastuzumab within the hinge (Figure 1B). Since the solution-phase kinetics of IgG single hinge cleavage by IdeS can be better controlled *in vitro *compared with the human MMPs tested in this study, the scIgG-T used for the *in vitro *characterization and *in vivo *efficacy studies was prepared by incubating trastuzumab with IdeS. The resultant scIgG-T was purified as previously reported [18].

The purity of scIgG-T was analyzed by SDS-PAGE separation under both nonreducing conditions (Figure 1C) and reducing conditions (Figure 1D). Under nonreducing/denaturing conditions, the intact trastuzumab was detected as a single band of ~150 kDa. The IdeS scIgG-T was detected as two major bands, with the upper band representing the single hinge cleaved intermediate (135 kDa band) and the lower band (~26 kDa) corresponding to the detached Fc monomer (Fc_(m)_) in the denaturing conditions (Figure 1C). A faint band below 135 kDa was visible and is most probably the (Fab)_2 _fragment that migrates as a 110 kDa band by SDS-PAGE under nonreducing conditions (Figure 1C). The proteolytic activity of IdeS is quite active to cleave at the hinge of the first heavy chain, but the hinge of the second heavy chain is relatively resistant to the IdeS proteolytic cleavage. As a result, a small fraction of the double-cleaved (Fab)_2 _fragment exists in the scIgG-T preparation.

Under reducing/denaturing conditions, the intact trastuzumab is separated into a heavy chain band (~50 kDa) and a light chain band (~25 kDa) (Figure 1D, right lane), whereas the IdeS single hinge cleavage of trastuzumab resulted in four fragments: the ~50 kDa intact heavy chain, the ~25 kDa light chain, and the two heavy chain proteolytic cleavage fragments (Fc_(m) _and scHC) migrated slightly above the light chain band (Figure 1D).

**Figure 2:**
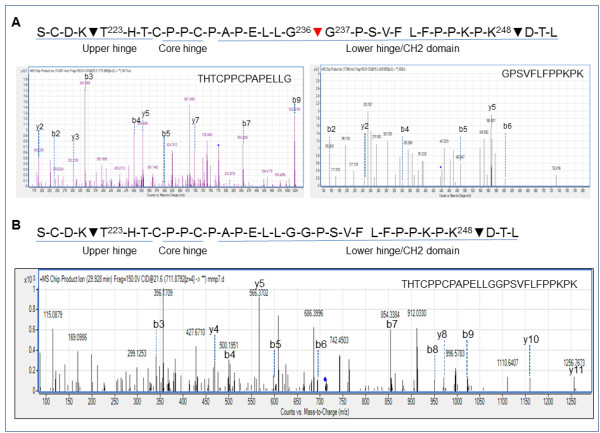
**Detection of single hinge cleaved trastuzumab and trastuzumab proteolytic cleavage using mass spectrometry**. **(A) **Sequence of the hinge region of trastuzumab showing the major trypsin cleavage sites (black arrows) and the IgG-degrading enzyme S (IdeS) cleavage site (red arrow). Bottom graph: liquid chromatography (LC)/mass spectrometry (MS)/MS analysis of trypsin-treated single hinge cleaved trastuzumab (scIgG-T), identifying the two peptides: THTCPPCPAPELLG and GPSVFLFPPKPK. Spectra are the MS2 spectra showing the b and y ions for the two peptides, confirming their sequences. **(B) **Sequence of the hinge region of trastuzumab. Black arrows, major trypsin cleavage sites. Bottom graph: LC/MS/MS analysis of trypsin-treated trastuzumab that identifies the peptide THTCPPCPAPELLGGPSVFLFPPKPK. Spectra are the MS2 spectra showing the b and y ions for the peptide, confirming its sequence.

Sequence analysis of the tryptic digestion products of scIgG-T using MS confirmed the IdeS cleavage site at G236^-/-^G237 (Figure 2A). In comparison, the tryptic digestion of intact trastuzumab showed only a hinge peptide from T223 to K248 as expected (Figure 2B).

### scIgG-T retains HER2 antigen engagement activity

**Figure 3:**
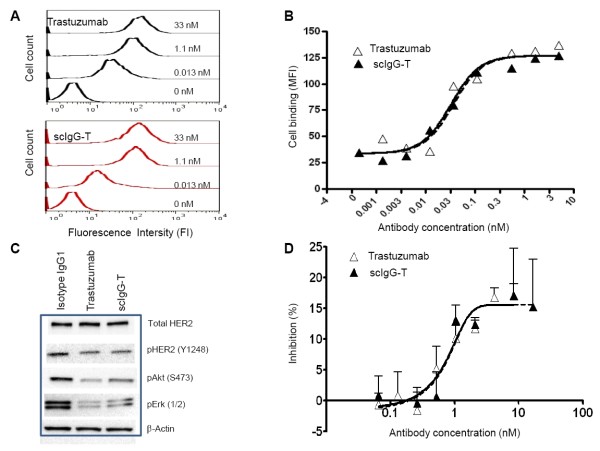
**Biological characterization of single hinge cleaved trastuzumab compared with the intact trastuzumab antibody *in vitro***. **(A) **Histograms of trastuzumab and single hinge cleaved trastuzumab (scIgG-T) binding to HER2 expressed on BT474 cells using a flow cytometer. **(B) **Concentration-dependent binding of trastuzumab and scIgG-T to HER2 expressed on BT474 breast cancer cells as measured by flow cytometer. Mean fluorescence intensity (MFI) is plotted against each antibody concentration (nM) on the *x *axis. **(C) **Effect of trastuzumab and scIgG-T on total HER2 expression, pHER2 (Y1289), pAKT (S473), and pErk1/2 in BT474 cells as determined by western blotting. **(D) **Inhibition of BT474 breast cancer cell proliferation by trastuzumab and scIgG-T (*n *= 4). Percentage of cell growth inhibition calculated as: (fluorescence signal of control group - signal of treatment group)/signal of control group×100.

To study the effect of single hinge cleavage on trastuzumab HER2 binding and downstream signaling, we carried out a series of *in vitro *experiments using purified scIgG-T. The histograms collected from a flow cytometer for the binding of trastuzumab and scIgG-T on BT474 high HER2 expression breast cancer cells showed a concentration-dependent increase of the fluorescence signal (Figure 3A). The concentration-dependent binding to BT474 cells showed overlapping sigmoidal curves when plotted with fluorescence signals against antibody concentrations, and the half-maximal effective concentration values for trastuzumab and scIgG-T were 0.27 nM and 0.31 nM, respectively (Figure 3B). The effects of trastuzumab and scIgG-T on HER2 signaling were detected by western blotting (Figure 3C). Similar to trastuzumab, scIgG-T decreased phosphorylation of pHER2 (Y1248), pAKT (S473) and pErk1/2 in BT474 cells (Figure 3C). There were minimal effects on total HER2 levels by either scIgG-T or the intact trastuzumab (Figure 3C). Consistent with inhibition of pAKT (S473) and pErk1/2, the intact trastuzumab and scIgG-T showed similar inhibition of cancer cell proliferation (Figure 3D). Collectively, these results indicated that a single cleavage of trastuzumab within the lower hinge did not impact antibody binding to the HER2 antigen, inhibition of downstream signaling, and inhibition of cell proliferation.

### scIgG-T reduced binding to Fc gamma receptors and diminished Fc-mediated ADCC activities

**Figure 4:**
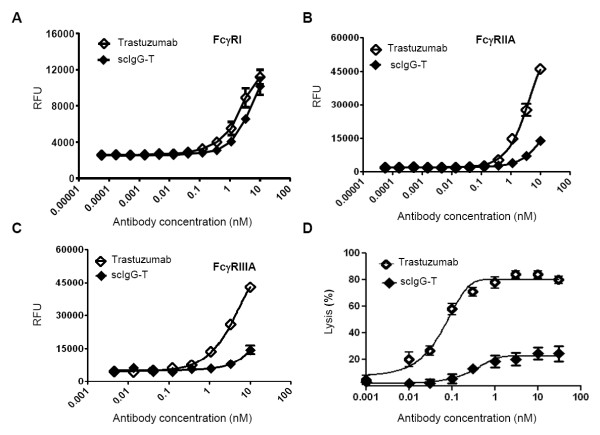
**Trastuzumab and single hinge cleaved trastuzumab binding to Fcγ receptors and antibody-dependent cellular cytotoxicity activity**. **(A) **Trastuzumab and single hinge cleaved trastuzumab (scIgG-T) binding to Fc gamma receptor (FcγR) I. **(B) **Trastuzumab and scIgG-T binding to FcγRIIA. **(C) **Trastuzumab and scIgG-T binding to FcγRIIIA. **(D) **Antibody-dependent cellular cytotoxicity (ADCC) activity induced by trastuzumab and scIgG-T using the high-HER2-expressing SKOV-3 ovarian cancer cells as target cells and human peripheral blood mononuclear cells (PBMC) as effector cells (*n *= 3). Cell index was compared after treatment of trastuzumab and scIgG-T for 24 hours. Percentage of cell lysis calculated as: (cell index of control group - cell index of treatment group)/cell index of control group×100. RFU, relative fluorescence units.

Fc gamma receptors (FcγRs) are expressed on diverse immune cell types and are important for antibody engagement of immune effector cell functions such as ADCC. Binding of the intact trastuzumab and scIgG-T to ADCC-activating receptors (FcγRI, FcγRIIA, and FcγRIIIA) was assessed using ELISA. Intact trastuzumab demonstrated binding to all three FcγRs in a concentration-dependent manner (Figure 4A to 4C). In contrast, scIgG-T had minimal binding to the FcγRIIA and FcγRIIIA receptors (Figure 4B,C). Binding to the high-affinity FcγRI was also reduced by scIgG-T, although the reduction was not as marked when compared with FcγRIIA and FcγRIIIA (Figure 4A). ADCC activities mediated by the intact trastuzumab and scIgG-T were determined using human PBMC as effector cells and the high HER2 expression SKOV-3 ovarian cancer cells as target cells. Cancer cell lysis mediated by the intact trastuzumab reached over 80% at a concentration as low as 1 nM; however, cell lysis induced by scIgG-T reached a 20% maximum at the same concentration (Figure 4D). The decrease in ADCC activity mediated by scIgG-T was consistent with its reduced binding affinity to FcγRs expressed on immune effector cells, most notably the loss of binding to FcγRIIIa that is known for its contribution to ADCC and is the main FcγR expressed on NK cells.

### scIgG-T had reduced anti-tumor efficacy in a mouse xenograft tumor model

**Figure 5:**
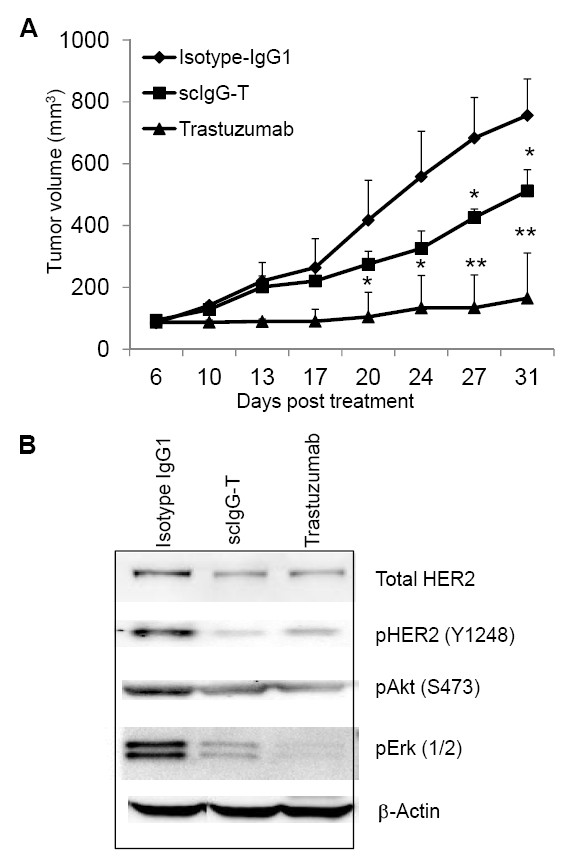
**Trastuzumab and single hinge cleaved trastuzumab tumor inhibition efficacy in breast cancer xenograft mouse model**. **(A) **BALB/c nu/nu mice (*n *= 5) were subcutaneously inoculated with 5×10^6 ^BT474 human breast cancer cells. Mice were treated with the antibodies at 5 mg/kg weekly for a total of five doses when tumors reached an average size of 100 mm^3^. Tumor sizes were measured and compared among the treatment groups. *P < 0.05 and **P < 0.005. **(B) **Tumor lysates were prepared from the frozen xenograft tumor tissues. Total HER2 expression, pHER2 (Y1248), pAKT (S473), and pErk1/2 were determined by western blotting. scIgG-T, single hinge cleaved trastuzumab.

The impact of single hinge cleavage on trastuzumab efficacy *in vivo *was determined by comparing tumor growth inhibition by the intact trastuzumab and scIgG-T in a mouse xenograft tumor model using high-HER2-expressing BT474 breast cancer cells. The antibody was dosed at 5 mg/kg and administered once weekly for 4 weeks (Figure 5A). Compared with the isotype IgG control groups, trastuzumab completely inhibited tumor growth (Figure 5A). However, the scIgG-T-treated mice showed significantly lower inhibition of tumor growth than that of trastuzumab with a maximum of ~30% inhibition of tumor growth when compared with the isotype control IgG (Figure 5A). To understand the mechanism of tumor growth inhibition by scIgG-T and trastuzumab, we conducted *ex vivo *studies to investigate the effect of trastuzumab and scIgG-T on HER2-mediated downstream signaling in xenograft tumors. Both intact trastuzumab and scIgG-T-treated tumor extracts showed downregulation of total HER2 levels, and inhibition of pHER2 (Y1248), pAKT(S473), and pErk1/2 when compared with tumors treated with the isotype control (Figure 5B). These results suggest that scIgG-T retained its HER2 antigen engagement and inhibitory effects on cancer cell signaling.

### scIgG-T reduced infiltration of immune effector cells in the mouse xenograft tumor

**Figure 6:**
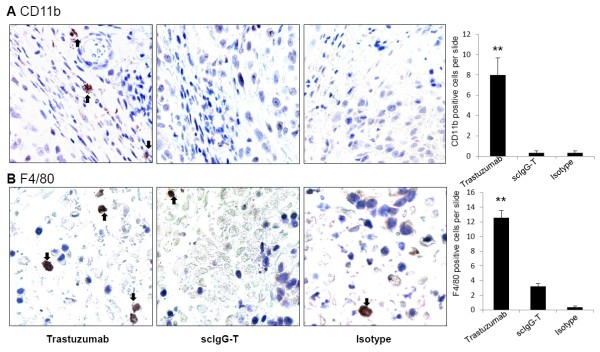
**Immune cell infiltration in xenograft tumors measured by immunohistochemistry**. Mouse xenograft tumors treated with trastuzumab, single hinge cleaved trastuzumab (scIgG-T), and the isotype control were fixed in 4% paraformaldehyde. Six immunohistochemistry slides were prepared from the paraffin-embedded tissues of two mice from each treatment group. Monocytes/macrophages were stained using the anti-mouse integrin αM/CD11b and anti-mouse F4/80 antibodies. **(A) **Representative images (×40) of CD11b-stained tumor tissue and positive-stained immune cells indicated by black arrows. Right bar graph: average number of CD11b-positive cells per tumor tissue slide (*n *= 6). Pairwise *t *test used to compare different treatment groups. **P < 0.005. **(B) **Representative images (×40) of F4/80-stained tumor tissues from different treatment groups. Right bar graph: Average number of positive stained cells detected per tissue slides (*n *= 6). Pairwise *t *test used to compare different treatment groups. **P < 0.005.

To study whether scIgG-T lost Fc-mediated immune cell effector function *in vivo*, we studied the effect of trastuzumab and scIgG-T on the infiltration of mouse immune effector cells in the xenograft tumors. Mouse immune cell infiltration was determined by immunohistochemistry using both anti-CD11b and F4/80 primary antibodies that can recognize infiltrated mouse macrophages and monocyte-derived immune cells. Trastuzumab-treated tumor tissues showed infiltrated immune cells when detected with both macrophage markers (Figure 6A,B). In contrast, tumor tissue treated with scIgG-T showed much fewer numbers of mouse macrophage infiltration and presented no difference from the tumor tissues treated with the isotype IgG control (Figure 6). Statistical analysis of the number of immune cells detected in tumor tissue slides in each group of three mice showed a significant increase of infiltrated immune cells in the intact trastuzumab-treated group (Figure 6, right bar graph). The reduced immune cell infiltration in scIgG-T treated tumors is consistent with the *in vitro *low ADCC activity mediated by scIgG-T (Figure 4D). These results imply that the decreased anti-tumor activity of scIgG-T *in vivo *is the result of reduced ADCC-mediated by immune effector cells.

## Discussion

The correlation between accumulation of proteinases, such as MMPs, in the tumor microenvironment and cancer progression and metastasis is well documented [31,33-37]. However, the role of MMPs in antibody hinge cleavage and the consequence of such antibody cleavage events in tumor microenvironment are unknown. Primary and acquired resistance to cancer therapeutic monoclonal antibodies such as trastuzumab in the clinic is one of the major challenges in cancer therapy [10,38]. Mechanisms resulting in refractory and acquired resistance to trastuzumab are poorly understood, and different models have been proposed including genetic mutations among key genes in the HER signaling pathway such as phosphatase and tensin homolog as well as upregulation of oncogenes such as cMET, epidermal growth factor receptor and HER3 [8,10,39]. The ability of MMPs to cleave IgG_1 _antibodies specifically in the lower hinge region *in vitro *suggests a possible link between MMP accumulation and loss of antibody Fc-mediated immune effector function in the tumor microenvironment. Using trastuzumab and high-HER2-expressing breast cancer cells, this study provided for the first time *in vitro *and *in vivo *experimental evidence that proteinase cleavage of trastuzumab at the lower hinge compromised its Fc-mediated immune effector functions such as ADCC *in vitro*, which resulted in reduced anti-tumor efficacy *in vivo*. We used the bacterial proteinase IdeS to prepare scIgG-T in our studies for two reasons. First, IdeS cleaves the IgG_1 _antibody with high specificity and generates a scIgG intermediate similar to IgG_1 _cleavage products by human MMPs. Second, IdeS cleaves the first strand rapidly and the second strand slowly, which presents a practical window for the isolation of homogeneous scIgG-T for *in vitro *and *in vivo *studies [14,18,40].

In this study, trastuzumab was readily cleaved *in vitro *by a panel of human MMPs (MMP-3, MMP-7, MMP-12, and MMP-13), which was consistent with previous reports that human IgG_1 _antibodies are susceptible to proteinase cleavage [14,15,18,20,21,40]. However, MMPs are complex proteinases and their proteolytic activities are tightly regulated *in vivo*, and *in vitro *assays may not reflect their *in vivo *activities [34]. It is noted that relatively high levels of MMP9 and MMP3 in the patient tumor sample were detected. Further studies are needed to establish the specificity and activity of the MMPs in the tumor microenvironment with respect to trastuzumab hinge cleavage.

To carry out downstream experiments, we prepared a large batch of scIgG-T by incubating trastuzumab with IdeS, which cleaves the antibody at the G236^-/-^G237 site. In comparison with trastuzumab, the IdeS-generated scIgG-T showed comparable functions related to HER2-mediated activities but exhibited reduced Fc-mediated ADCC activity *in vitro*. A previous report demonstrated that a single hinge cleaved IgG intermediate had a complete loss of ADCC function *in vitro *[18], whereas the scIgG-T tested in this study demonstrated significantly reduced ADCC but not a complete loss of function. One potential explanation for this discrepancy was that different methods were used for ADCC detection. The ADCC assay reported in this study was monitored over the course of 120 hours, whereas the ADCC assay from the previous report was carried out for 2 hours. Numerous studies indicate that cell lysis in a 2-hour ADCC assay is mediated by NK cells alone [41,42], and the only activating FcγR expressed by NK cells is FcγRIIIa [43]. The complete loss of function seen in a 2-hour ADCC assay is therefore consistent with the loss of binding to FcγRIIIa. When ADCC assays are performed for time periods exceeding 12 hours, myeloid linage cells can also mediate *in vitro *ADCC [44]. The detection of ADCC activity with scIgG-T in the present report, albeit at a much reduced level relative to the intact version of IgG_1_, perhaps reflects some degree of recruitment of myeloid-mediated ADCC activity in addition to NK function. This low level of ADCC activity is consistent with the ability of scIgG-T to bind to the high-affinity FcγRI expressed in the monocyte population.

The mechanisms of action for trastuzumab can be largely grouped into two categories: one is associated with antagonizing HER2 signaling through Fab region of the IgG_1 _molecule, and the other is through Fc-mediated immune effector cell functions. The partial anti-tumor efficacy conferred by scIgG-T in the mouse xenograft model can be attributed to the direct HER2 engagement by the Fab region of scIgG-T, as indicated by downregulation of HER2 in tumor tissues and inhibition of pHER2 (Y1248), pAKT (S473), and pErk1/2. These results are consistent with previous findings that trastuzumab mechanisms of action include inhibition of HER2 downstream signaling [8,39,45] and downregulation of HER2 receptor level [46].

Because scIgG-T showed diminished Fc-mediated ADCC *in vitro*, it is expected that scIgG-T will be less effective in engaging immune cell-mediated cancer cell killing *in vivo*. As an IgG_1 _isotype antibody, trastuzumab mediates ADCC by recruiting immune effector cells to HER2-overexpressing tumor cells [25]. Xenograft studies in FcγR knockout mice showed that interaction with the ADCC activation receptor (FcγRIII) is essential for the efficacy of trastuzumab [3]. Patients with the higher binding affinity FcγRIIIa-158V/V genotype showed an improved response to trastuzumab than patients with the lower affinity FcγRIIIa-158 F/F and F/V genotypes, again suggesting that Fc-mediated effector function plays a key role in the clinical efficacy of trastuzumab [4]. Even though the correlation between FcγRIIIa polymorphisms and outcome in trastuzumab-treated cancer patients was not confirmed in a recent clinical study [47], the important role of FcγRIIIa in Fc-mediated immune function is not in dispute. The reduced anti-tumor efficacy by scIgG-T strongly supports that Fc-mediated effector function is a key mechanism of action for *in vivo *efficacy of trastuzumab. The significantly reduced immune cell infiltration shown in scIgG-T-treated mouse xenograft tumor as compared with that treated with intact trastuzumab again supports the notion that scIgG-T has compromised Fc effector function due to lack of engagement of immune cells at the tumor site. Mouse macrophages are phagocytic and express CD11b and F4/80 [48]. Macrophage recruitment to the tumor site has been documented in a PyMT mouse breast cancer model [48,49]. Even though the nude mouse used in the xenograft tumor model is believed devoid of T cells, the results in the present study clearly indicate that monocyte-derived immune cell infiltration to tumor tissue occurs in the nude mouse tumor model when treated with trastuzumab. The association of reduced anti-tumor efficacy by scIgG-T with the decreased number of infiltrated immune cells further suggests that infiltrated mouse immune cells in xenograft tumor tissue are capable of mediating anti-tumor activities when high HER2 expression cancer cells are treated with trastuzumab. This finding is consistent with previous reports on the correlation of immune effector cell infiltration and trastuzumab efficacy in the clinic [24,25].

**Figure 7:**
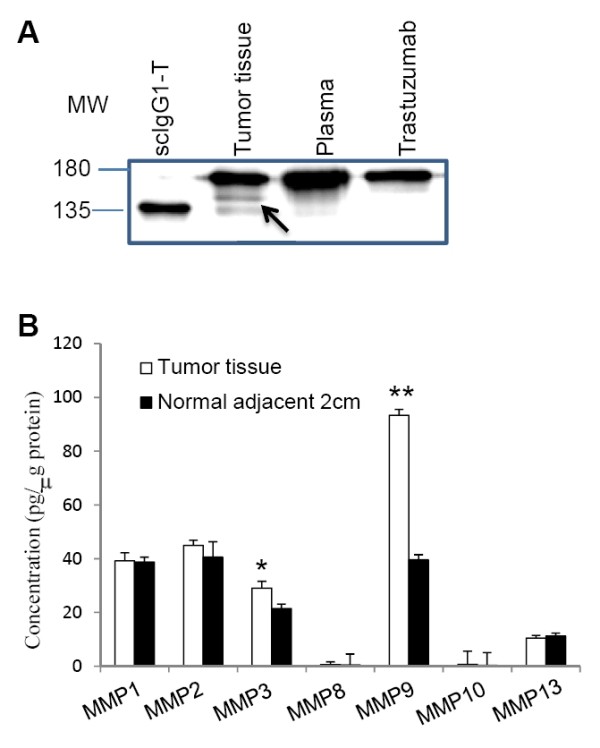
**Detection of single hinge cleaved trastuzumab and matrix metalloproteinases in breast cancer patient samples**. **(A) **Same amounts of enriched IgGs from each sample were separated by SDS-PAGE under nonreducing/denaturing conditions. Arrow, single hinge cleaved trastuzumab (scIgG-T) as referenced to standard preparation in the right lane. **(B) **Detection of matrix metalloproteinase (MMP) expression in breast cancer patient tumor tissues and normal adjacent 2 cm tissue using a reverse protein array method (*n *= 4). *P < 0.05 and **P < 0.005. MW, molecular weight.

This study showed that trastuzumab can be readily cleaved by MMPs in the lower hinge *in vitro *and that scIgG-T showed much reduced anti-tumor efficacy in comparison with the intact trastuzumab *in vivo*. Further investigation is necessary to determine whether the hinge cleavage of trastuzumab occurs in the tumor microenvironment in the clinical setting. It is interesting to note that a patient who was diagnosed with HER2-overexpressing breast cancer and was under adjuvant treatment with trastuzumab and chemotherapy for 6 months showed evidence of hinge cleavage of trastuzumab in the tumor tissue (Figure 7A). More importantly, single hinge cleavage of trastuzumab was detected in the tumor tissue but was not visible in the plasma sample where other host IgG antibody is abundant (Figure 7A). Expression of MMPs (MMP-1, MMP-2, MMP-3, MMP-8, MMP-9, MMP-10, and MMP-13) was also analyzed using a reverse-phase protein array. Among the seven MMPs tested, both MMP-3 and MMP-9 were elevated in samples from the tumor site when compared with that from the adjacent normal tissues (Figure 7B).

While the extent of the hinge cleavage of trastuzumab in cancer patients during the antibody treatment is yet to be established in more patient samples, our data demonstrated that the cleavage of trastuzumab and generation of scIgG-T can occur in the tumor microenvironment. Furthermore, we hypothesize that hinge cleavage from trastuzumab to scIgG-T in the tumor microenvironment may lead to reduced anti-tumor efficacy by weakening the antibody Fc interaction with immune effector cells. However, the extent of the hinge cleavage of trastuzumab in breast cancer patients is yet to be established in a large patient cohort. Further investigation in clinical settings is warranted to determine whether single hinge cleavage represents a novel mechanism for cancer cells to invade immune functions mediated by trastuzumab *in vivo*.

## Conclusion

Studies of resistance mechanisms of trastuzumab have been focused on genetic mutations in the HER2 signaling pathways and little is known on the role of immune evasion in the development of trastuzumab resistance. This study demonstrated that trastuzumab can be cleaved by MMPs within the lower hinge and that the hinge cleaved trastuzumab (scIgG-T) showed a significantly reduced anti-tumor efficacy due to the weakened immune effector function, including ADCC. Taken together, the results in this study suggest that hinge cleavage from intact trastuzumab to scIgG-T in the tumor microenvironment may lead to reduced anti-tumor efficacy by weakening the antibody's ability to mediate immune effector functions. Further studies are warranted to understand the significance of single hinge cleavage on trastuzumab efficacy in the clinic.

## Abbreviations

ADCC: antibody-dependent cellular cytotoxicity; AKT: protein kinase B; ELISA: enzyme-linked immunosorbent assay; Erk: extracellular-signal-regulated kinase; Fab: fragment antigen binding; Fc: fragment crystallizable; Fc_(m)_: Fc monomer; FcγR: Fc gamma receptor; HER: human epidermal growth factor receptor; IdeS: immunoglobulin G-degrading enzyme S; IgG: immunoglobulin G; MMP: matrix metalloproteinase; MS: mass spectrometry; NK: natural killer; PBMC: human peripheral blood mononuclear cells; PBS: phosphate-buffered saline; scIgG-T: single hinge cleaved trastuzumab.

## Competing interests

This study was partially funded by a grant from Janssen R&D, LLC. RJB, AO, WRS, and REJ are employees of Janssen R&D, LLC. The remaining authors declare that they have no competing interests.

## Authors' contributions

XF participated in the study design and writing the Materials and methods sections, and conducted mouse tumor xenograft studies and *in vitro *ADCC and western blotting assays. RJB contributed to scIgG-T preparation, study design, results interpretation and editing the manuscript. MF conducted MS analysis and prepared mass spectra graphs. HD conducted *in vitro *cancer cell proliferation assays and FcγR assays. AO conducted hinge cleavage of trastuzumab by human MMPs and IdeS proteinase. AG is the medical investigator for the approved institutional review board protocol and consented patients, and provided patient clinical samples and information. WPD supervised and provided expertise in MS. WRS was involved in study design and manuscript review. REJ participated in the study design, supervised scIgG-T preparation and edited the manuscript. NZ designed experiments, supervised *in vitro *and *in vivo *assay development and wrote the manuscript. ZA conceived the study, interpreted data, and drafted the manuscript. All authors read and approved the final manuscript.
